# Dose Effect of Poractant Alfa in Neonatal RDS: Analysis of Combined Data from Three Prospective Studies

**DOI:** 10.3389/fped.2020.603716

**Published:** 2020-11-24

**Authors:** Barbara Królak-Olejnik, Roman Hożejowski, Tomasz Szczapa

**Affiliations:** ^1^Department of Neonatology, Wroclaw Medical University, Wroclaw, Poland; ^2^Medical Department, Chiesi Poland, Warsaw, Poland; ^3^Department of Neonatology, Biophysical Monitoring and Cardiopulmonary Therapies Research Unit, Poznan University of Medical Sciences, Poznan, Poland

**Keywords:** surfactant, poractant alfa, respiratory distress syndrome, preterm neonate, dose, dose—response, neonatal outcome, less invasive surfactant administration (LISA)

## Abstract

**Aim:** To evaluate the effect of the initial dose of poractant alfa on clinical outcomes in neonatal respiratory distress syndrome (RDS) and to assess adherence to treatment guidelines recommending a dose of 200 mg/kg.

**Methods:** Records of neonates who received poractant alfa with a less invasive technique (LISA) or with the INtubate-SURfactant-Extubate (INSURE) technique were retrieved from the aggregated datasets of three prospective RDS studies conducted between 2015 and 2019. The impact of poractant dose on neonatal outcomes was analyzed by multivariate logistic regression. The primary endpoint was the need for early (<72 h of life) mechanical ventilation (MV). Typical complications of prematurity and the need for surfactant retreatment were secondary endpoints. Deviation from the 200 mg/kg dose of surfactant was a measure of compliance with the treatment guidelines. As a complementary analysis, the rates of adverse outcomes were compared for infants receiving high (200 mg/kg ±10%) and low (100 mg/kg ±10%) doses of poractant.

**Results:** Of 994 eligible infants, 574 received poractant alfa with LISA, and 420 received poractant with INSURE. A logistic regression model using data from all 994 infants showed that the surfactant dose had a significant effect on reducing the need for MV and retreatment; the respective odds ratios were 0.92 (95% CI: 0.90–0.95) and 0.93 (95% CI: 0.90–0.96) per 10-mg/kg dose increment of poractant alfa. This dose effect was observed across all gestational age ranges and in infants treated with LISA. In newborns treated with INSURE, the dose of surfactant only influenced the rates of retreatment (*p* = 0.036) but not MV (*p* = 0.170). No impact on other neonatal outcomes was observed. In the subset of infants who received high (*N* = 502) and low (*N* = 58) doses of poractant, the high-dose group had lower rates of MV (34 vs. 48%, *p* = 0.042) and lower rates of retreatment (11 vs. 21%, *p* = 0.045). Surfactant underdosage increased with gestational age and ranged from a minimum of −3 mg/kg in <26 weeks to a maximum of −23.5 mg/kg in >32 weeks.

**Conclusions:** The initial dose of poractant alfa significantly impacts the need for invasive ventilation and retreatment. More mature newborns are at a greater risk of underdosing.

## Introduction

European guidelines for the management of respiratory distress syndrome (RDS) specify a minimum dose of surfactant equal to 100 mg/kg. However, the guidelines recommend a high dose of 200 mg/kg poractant alfa as being more effective than a low dose (100 mg/kg) of either poractant alfa or beractant (1). Despite available evidence and recommendations, a high dose is not always followed in everyday practice. As shown in recently published studies, achieving a dose of 200 mg/kg in daily practice is questionable, and underdosing is common (2, 3). Irrespective of this, some centers still use a low dose (100 mg/kg).

Prospective, randomized trials showing more favorable treatment outcomes with a high dose of surfactant were carried out in 2004 (4), 2010 (5), and 2012 (6). Surfactant was then administered via an endotracheal tube, and the patients were often subjected to subsequent mechanical ventilation. However, treatment paradigms have evolved in recent years, and the strategy of choice is currently less invasive surfactant administration (LISA) in newborns supported by noninvasive ventilation. For this reason, it seemed advisable to verify previous findings regarding the high dose in the newer cohorts treated predominantly with noninvasive respiratory support and LISA.

Based on a relatively recent sample of premature infants treated in accordance with current strategies, we aimed to verify the impact of the initial dose of surfactant on the need for mechanical ventilation (MV) and the incidence of selected neonatal outcomes. In addition, the adverse outcome rates for infants receiving high and low doses of poractant alfa were compared. As a secondary objective, we assessed adherence to the recommended dosage of 200 mg/kg surfactant in daily practice.

## Materials and Methods

To evaluate the dose effect of poractant alfa, datasets from three prospective cohort studies conducted in Poland between 2015 and 2019 were combined. These studies included a total of 1,654 premature infants with RDS admitted to level-3 NICUs across the country. The reason for aggregating study datasets was to obtain a reasonable number of observations on low-dose poractant alfa, which is rarely used in Poland.

Detailed design and the results of the three studies have been reported elsewhere (7–9). Table 1 summarizes the studies included in the analysis. Of note, all studies were prospective and noninterventional, and the protocols did not require interference with standard therapeutic regimens in participating centers. All three studies obtained prior approval from the Ethics Committee of Warsaw Medical University in accordance with the affiliation of the study principal investigator. Parents/legal guardians gave written consent to all diagnostic and therapeutic procedures in compliance with local legislation and practices.

**Table 1:** Summary of three neonatal RDS cohort studies combined in the analysis.

	**Study 1**	**Study 2**	**Study 3**
	**Borszewska-Kornacka et al. (7)**	**Gulczyńska et al. (8)**	**Szczapa et al. (9)**
Sample size	Total = 986	Total = 394	Total = 500
Key enrollment criteria	• GA ≤ 32 weeks	• GA <30 weeks	• GA ≤ 36 weeks
	• RDS	• RDS	• RDS
	• Treatment with SF	• CPAP as initial respiratory support	• LISA
Study period	January 2015–December 2015	March 2017–September 2018	February 2018–March 2019
**Surfactant**			
None	–	157 (39.8%)	–
Poractant alfa	918 (93.1%)	235 (59.6%)	491 (98.2%)
Beractant	44 (4.5%)	2 (0.5%)	9 (1.8%)
Missing data	24 (2.4%)	–	–
**Initial dose of poractant alfa (mg/kg)**			
Median (IQR)	167 (133–196)	185 (145–200)	193 (158–200)
Range	57–250	64–343	41–267
**RDS management**^**§**^			
Primary intubation + SF + MV	571	–	–
**Primary NIV**			
SF (LISA or INSURE)	330	173	491
SF (MV)	–	61	–
**Poractant alfa with LISA or INSURE**	***n*** **=** **330**	***n*** **=** **173**	***n*** **=** **491**
Initial dose 100 mg/kg^#^	33/330 (10%)	9/173 (5.2%)	16/491 (3.3%)
Initial dose 200 mg/kg^#^	130/330 (39.4%)	84/173 (48.6%)	288/491 (58.7%)

§ *Data on the mode of surfactant administration were unavailable for 17 patients in Study 1 and 1 patient in Study 2*.

# *Including ±10% deviation*.

*GA, gestational age; SF, surfactant; CPAP, continuous positive airway pressure; MV, mechanical ventilation; NIV, noninvasive ventilation; LISA, less invasive surfactant administration; INSURE, intubation-surfactant-extubation*.

Eligible patients were premature infants who did not need primary intubation and were started on noninvasive ventilation shortly after birth and required surfactant in the further course of treatment. Surfactant was either administered with LISA or (less often) with the INSURE technique (INtubate-SURfactant-Extubate). The use of INSURE implied extubation within a maximum of 60 min; otherwise, the infant was classified as receiving MV.

To assess the dose effect of surfactant, a multivariate logistic regression analysis was performed, with neonatal outcomes as dependent variables, whereas the surfactant doses from all infants, patient demographics, and perinatal and respiratory parameters were included as covariates. For the complementary analysis, which was a direct comparison of the effects of high and low doses of surfactant, infants receiving 100 mg/kg ± 10% poractant alfa were categorized as the low-dose group, and those receiving 200 mg/kg ± 10% were categorized as the high-dose group.

### Study Endpoints

The endpoints studied included the need for invasive ventilation in the first 72 h of life, in-hospital mortality, bronchopulmonary dysplasia (BPD), intraventricular hemorrhage (IVH), retinopathy of prematurity (ROP), persistent ductus arteriosus (PDA), periventricular leukomalacia (PVL), and the need for surfactant retreatment.

Deviation from the 200-mg/kg dose of surfactant was a measure of compliance with the treatment guidelines.

### Statistics

Patient data were tested for normality with the D'Agostino–Pearson test and presented as the means (standard deviations, SDs) or medians (interquartile ranges, IQRs) as appropriate.

The multiple logistic regression model used the entire range of poractant alfa doses, which were treated as a continuous variable. The odds ratios for adverse outcomes were calculated per 10 mg/kg increase in poractant alfa dose. Taking the heterogeneity of the pooled studies, a regression analysis was replicated across gestational age ranges: 24–28, 29–32, and >32 weeks. Separate models were also computed for patients receiving surfactant with LISA and INSURE.

The Fisher exact test or chi-square test was used in the complementary comparison of the adverse outcome rates between the high- and low-dose groups. All tests were two-tailed, and alpha = 0.05 was considered significant.

## Results

Of 1,654 babies in the combined dataset, we identified 994 infants who did not require primary mechanical ventilation and were given surfactant with LISA (57.7%) or INSURE (42.3%). The clinical characteristics of the analyzed cohort of 994 neonates stratified by assignment to individual studies are presented in Table 2.

**Table 2:** Clinical characteristics of the pooled study cohort (*N* = 994).

	**All**	**Study 1**	**Study 2**	**Study 3**
	***N* = 994**	***N* = 330**	***N* = 173**	***N* = 491**
Sex (male)	539 (54%)	179 (54%)	93 (54%)	267 (54%)
Gestational age (weeks)	29 (27.6–31)	29 (27.4–30.3)	28.1 (27–29)	30 (28–32)
Antenatal steroids	824 (83%)	282 (85%)	158 (91%)	384 (78%)
Birth weight (g)	1210 (960–1,521)	1200 (960–1,455)	1050 (900–1,265)	1330 (990–1,700)
C-section	872 (88%)	275 (83%)	152 (87%)	445 (91%)
Apgar 5 min	8 (7–8)	7 (7–8)	7 (7–8)	8 (7–8)
Caffeine citrate	977 (98%)	318 (96%)	172 (99%)	487 (99%)
FiO_2_ prior to SF	0.40 (0.35–0.50)	0.40 (0.30–0.50)	0.40 (0.34–0.46)	0.40 (0.35–0.50)
SpO_2_/FiO_2_ prior to SF	222 (178–280)	227 (184–280)	225 (184–266)	212 (167–249)
Time to SF (h)	1.6 (0.7–4.8)	1.0 (0.5–3.5)	1.2 (0.6–3.1)	2.1 (0.8–6.8)
SF dose (mg/kg)	185 (145–200)	167 (133–196)	185 (145–200)	192 (158–200)

*Data are numbers (%) or medians (IQRs). SF, surfactant; SpO_2_, blood oxygen saturation; FiO_2_, fraction of inspired oxygen*.

### Dose Effect of Surfactant

**Figure 1:**
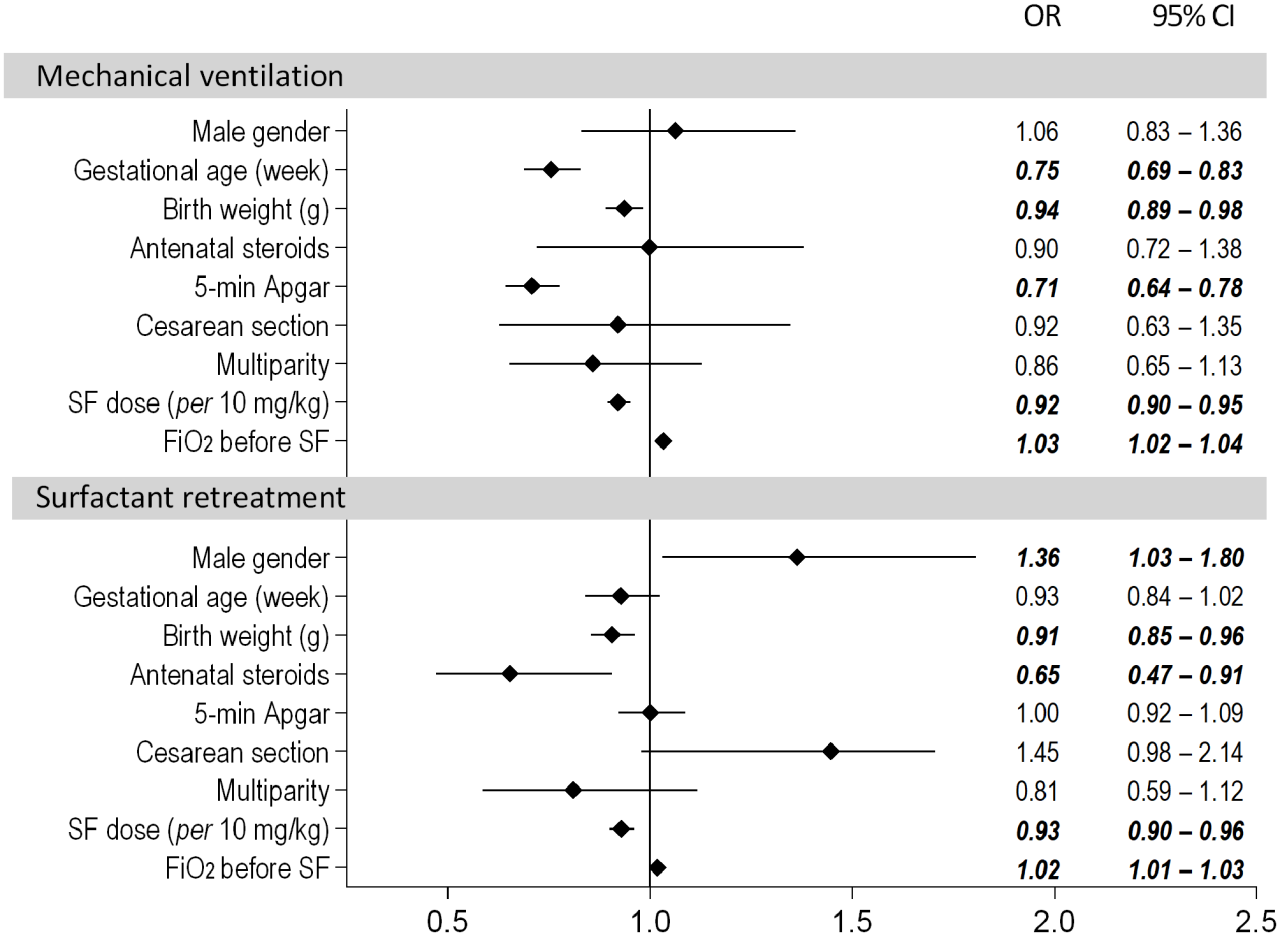
Results of the multivariate logistic regression model assessing the need for mechanical ventilation and surfactant retreatment. The dose of poractant alfa was used as a continuous variable.

In the multivariate logistic regression model covering the entire dose range, the risk of MV in the first 3 days of life was significantly influenced by the surfactant dose. The odds of MV were decreased by ~8% per 10-mg/kg increment of surfactant (OR = 0.92, 95% CI: 0.90–0.95). The risk of MV also decreased with higher gestational age, birth weight and 5-min Apgar score (Figure 1). The risk of MV was increased with a higher fraction of inspired oxygen (FiO_2_) prior to the administration of surfactant (OR = 1.03, 95% CI: 1.02–1.04).

Antenatal steroids had the strongest impact on the need for repeated doses of surfactant and reduced the odds of retreatment by ~35% (OR = 0.65, 95% CI 0.47–0.91). The risk also depended on surfactant initial dose; the odds of retreatment decreased by ~7% with every additional 10 mg/kg surfactant (OR = 0.93, 95% CI: 0.90–0.96).

Replication of the multivariate regression model across gestational age ranges (24–28, 29–32, and >32 weeks) confirmed that the surfactant dose was a significant variable affecting the need for MV <72 h and retreatment (Table 3). Subgroup analyses stratified by the mode of surfactant administration yielded a significant impact of surfactant dose on MV and retreatment in LISA-treated babies (*p* < 0.001). With INSURE, a dose effect was found for the incidence of retreatment (*p* = 0.036), but not for MV (*p* = 0.170).

**Table 3:** ORs for poractant alfa as a continuous covariate in the multiple logistic regression models evaluating subgroups; the remaining covariates were sex, gestational age, birth weight, antenatal steroids, 5-min Apgar, C-section, multiparity, and FiO_2_ before SF.

		**Mechanical ventilation** **<** **72 h**		**Surfactant retreatment**	
	***N***	OR^*^ **(95% CI)**	***p*-value**	OR^*^ **(95% CI)**	***p*-value**
All	994	0.92 (0.90–0.95)	<0.001	0.93 (0.90–0.96)	<0.001
LISA	574	0.89 (0.84–0.93)	<0.001	0.88 (0.82–0.94)	<0.001
INSURE	420	0.96 (0.91–1.02)	0.170	0.92 (0.84–0.99)	0.036
24–28 weeks	437	0.93 (0.88–0.98)	0.014	0.93 (0.86–1.01)	0.090
29–32 weeks	435	0.93 (0.88–0.98)	0.015	0.92 (0.85–0.99)	0.022
>32 weeks	122	0.85 (0.75–0.95)	0.004	0.80 (0.67–0.92)	0.004

* *per 10-mg/kg increment of poractant alfa*.

### High vs. Low Dose

A direct comparison of the effects of high and low doses of poractant alfa included 502 infants in the high-dose group and 58 infants in the low-dose group. Baseline characteristics showed no significant differences in demographics, clinical variables or oxygenation status prior to surfactant administration (Table 4).

**Table 4:** Clinical characteristics of the high- and low-dose groups.

	**High-dose SF**	**Low-dose SF**	***P*-value**
	**(*N* = 502)**	**(*N* = 58)**	
Sex (male)	275 (55%)	33 (57%)	0.867
Gestational age	29 (27–31)	29 (28–30)	0.761
Antenatal steroids	431 (86%)	46 (81%)	0.356
Birth weight (g)	1170 (956–1,477)	1200 (1,110–1,289)	0.347
C-section	439 (88%)	47 (81%)	0.212
Apgar 5 min	8 (7–8)	8 (7–8)	0.393
Caffeine citrate	499 (99.4)	55 (96.5)	0.084
FiO_2_ prior to SF	0.4 (0.35–0.5)	0.4 (0.35–0.5)	0.594
SpO_2_/FiO_2_ prior to SF	218 (176–260)	225 (180–266)	0.788
Time from birth to SF (h)	1.67 (0.68–3.9)	1.34 (0.62–7.56)	0.827
SF dose (mg/kg)	199 (193–202)	100 (95–104)	<0.0001

*Data are numbers (%) or medians (IQRs). SF, surfactant; SpO_2_, blood oxygen saturation; FiO_2_, fraction of inspired oxygen*.

Patients receiving a high dose of surfactant required invasive ventilation significantly less frequently during the first 3 days of life than those receiving a low dose of surfactant (34.1 vs. 48.3%, *p* = 0.042; Table 5). However, in babies requiring MV, its duration did not differ significantly between the groups. The need for retreatment was approximately twice as low for neonates treated with 200 mg/kg surfactant compared to those receiving 100 mg/kg surfactant (11.2 vs. 21.4%; *p* = 0.045). No significant differences were found for the other endpoints.

**Table 5:** Outcomes comparison in the high- and low-dose groups.

	**High-dose SF (*N* = 502)**	**Low-dose SF (*N* = 58)**	***P*-value**	**OR (95% CI)**
In-hospital mortality	25 (5%)	1 (1.7%)	0.505	3.00 (0.40–22.60)
BPD				
Mild	114 (23.5%)	10 (18.5%)		
Moderate	48 (9.9%)	4 (7.4%)	0.776	1.39 (0.75–2.56)^*^
Severe	17 (3.5%)	2 (3.7%)		
IVH	137 (27.5%)	22 (38.6%)	0.089	0.60 (0.34–1.07)
IVH grade 3 or 4	31 (6.2%)	3 (5.3%)	0.996	1.19 (0.35–4.04)
ROP requiring treatment	61 (12.3%)	5 (8.9%)	0.663	1.43 (0.55–3.72)
PVL	23 (4.7%)	3 (5.4%)	0.740	0.86 (0.25–3.00)
PDA requiring treatment	89 (17.9%)	7 (12.3%)	0.357	1.56 (0.68–3.56)
Surfactant retreatment	55 (11.2%)	12 (21.4%)	0.045	0.46 (0.23–0.93)
MV within 72 h of birth	171 (34.1%)	28 (48.3%)	0.042	0.55 (0.32–0.96)
Duration of MV (days), median IQR	6 (2-13.5)	4 (1.8-8)	0.152	–

* *OR calculated for the overall BPD incidence, regardless of the degree of severity*.

*Data were missing (not reported) in the following number of patients: Low-dose group: BPD = 4, IVH = 1, ROP = 2, PVL = 2, PDA = 1, surfactant redosing = 2; high-dose group: mortality = 2, BPD = 17, IVH = 4, ROP = 5, PVL = 8, PDA = 6, surfactant redosing = 10. Percentages were calculated relative to the number of patients with known outcomes*.

### Dose Adherence to Treatment Guidelines

In the entire cohort of 994 newborns treated with primary noninvasive ventilation and poractant alfa, the high-dose group accounted for 50.5%, and the low-dose group accounted for 5.8% of patients. Fifty-three infants (5.3%) received <100 mg/kg ±10% surfactant, 32 (3.2%) received more than 200 mg/kg ± 10%, and 349 (35.1%) received an amount of surfactant between that of the low and high doses.

**Figure 2:**
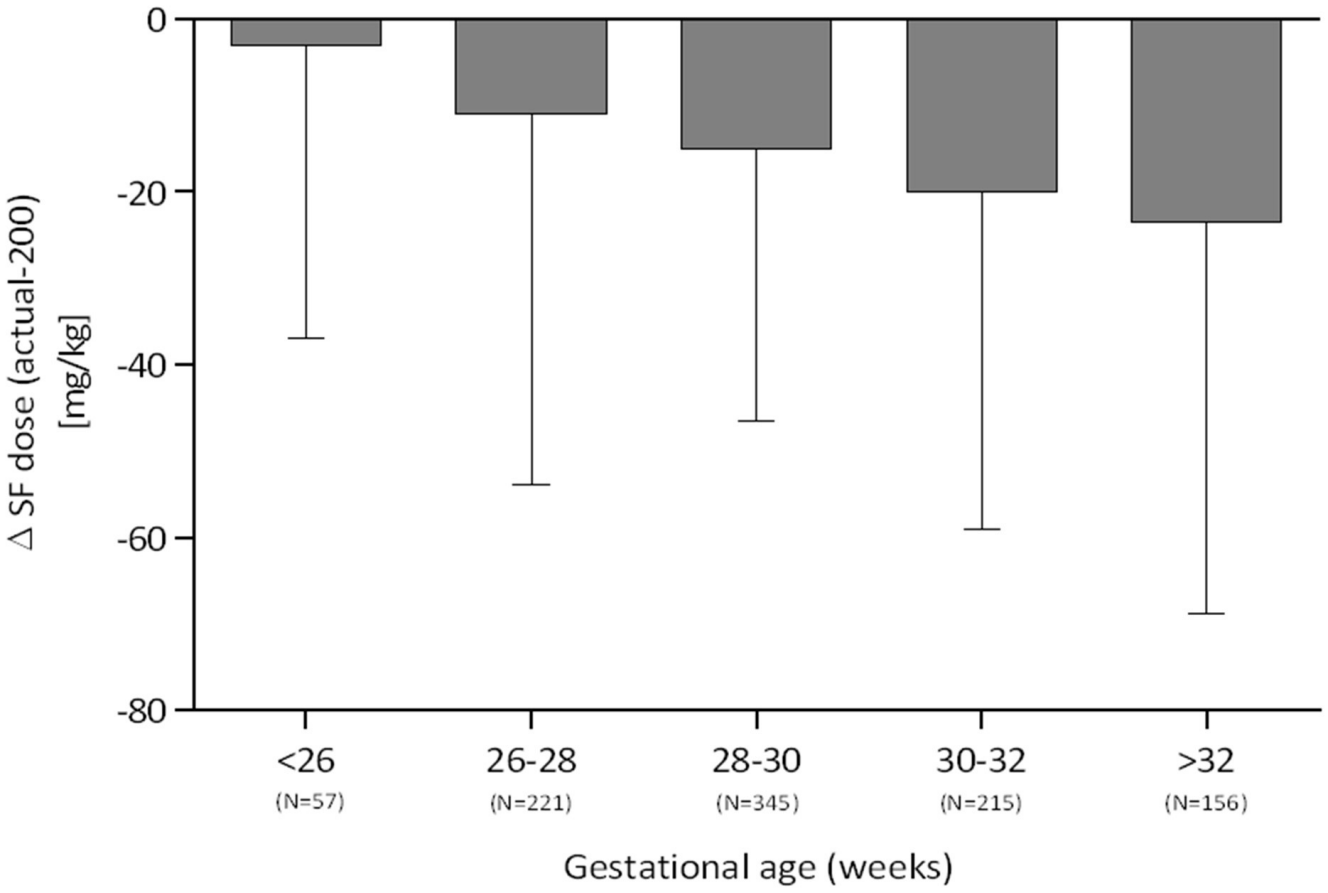
Deviation from the recommended dose of 200 mg/kg surfactant stratified by gestational age (median, IQR).

The actual dose of poractant alfa was consistent with the recommended 200 mg/kg dose only for most premature infants <26 weeks gestation (median deviation −3 mg/kg). As shown in Figure 2, median underdosing increased with gestational age and equaled −11 mg/kg for 26–28 weeks, −15 mg/kg for 28–30 weeks, −20 mg/kg for 30–32 weeks, and −23.5 mg/kg for >32 weeks.

## Discussion

Administration of surfactant is often needed to compensate for its deficit and relatively slow synthesis in preterm infants (10, 11). Thus, using the optimal dose may have an important role in the management of RDS. We have shown the effect of poractant alfa dose on selected clinical outcomes in the present analysis of a large merged cohort of preterm infants treated over the last 5 years.

The initial dose of surfactant had a significant impact on the need for invasive ventilation. This finding has important practical implications, given that the use of noninvasive ventilation techniques has seen a large rise in recent years. Measures that increase the likelihood of success with CPAP and thereby reduce the need for mechanical ventilation might potentially contribute to a decrease in BPD prevalence. However, differently from what has been previously shown in the literature (12), we did not see an effect on BPD in our study. One explanation could be that the number of babies receiving a low dose was very small relative to the number receiving a high dose. Another reason might be that BPD is a disease with multifactorial etiology. While mechanical ventilation and hyperoxia have a strong association with the pathogenesis of BPD, there are numerous pre- and postnatal factors contributing to the lung injury resulting in BPD (13). The use of novel techniques such as volume-targeted ventilation, servo-controlled oxygen algorithms, and inhaled NO could have already optimized the potential impact of ventilation on the lungs. Moreover, the currently observed BPD (“new BPD”) also occurs in preterm newborns who may have received little to no ventilation assistance (14, 15).

Another factor that significantly influenced the risk of MV was the FiO_2_ level prior to surfactant. This observation supports the significance of the appropriate FiO_2_ threshold for surfactant administration. The median FiO_2_ of 0.40 before surfactant was in the range reported in other publications, e.g., the study by Janssen et al. (16) from 2014 to 2016, where an FiO_2_ of 0.37 was recorded in successful LISA patients and an FiO_2_ of 0.44 was recorded in failed LISA patients. In the Spanish cohort from 2013 to 2015, the mean FiO_2_ was 0.47 (2).

The shift toward a lower FiO_2_ requirement indicative of surfactant therapy regardless of gestational age was noted in the last update of the European RDS Guidelines (1). The established threshold of FiO_2_ = 0.30 reflected increased knowledge regarding CPAP failure predictors (8, 17). Our findings on the role of FiO_2_ add to the body of evidence that supports the early use of surfactants.

The initial dose of poractant alfa had significant impact on the retreatment rates. This is consistent with the findings of earlier studies. In a prospective trial investigating the effects of porcine surfactant on pharmacokinetics and gas exchange, more babies needed the second dose of surfactant when treated with a low dose (70 vs. 28.6%) (10). Additionally, a retrospective review from New Zealand revealed that the proportion of patients requiring redosing was twice as high in infants receiving 100 vs. 200 mg/kg (18). Pharmacokinetic data may explain the connection between the retreatment rate and surfactant dose. The half-life of desaturated phosphatidylcholine (DSPC), the phospholipid primarily responsible for lowering alveolar surface tension, is significantly longer with the initial dose of 200 mg/kg porcine surfactant compared to 100 mg/kg (32 ± 19 vs. 15 ± 15 h; *p* = 0.002) (10).

In contrast to some of the earlier studies and the Cochrane review comparing animal-derived surfactants, we found no dose-related effects on mortality or complications of preterm birth, such as BPD, PDA, or IVH (4, 12, 15, 17). Nonetheless, superior respiratory effects of higher poractant doses were previously reported in prospective and retrospective studies. Cloete et al. (18) have shown greater reductions in FiO_2_, lower rates of pneumothorax, and a trend toward shorter mechanical ventilation in a retrospective analysis that included 256 preterm infants from New Zealand. A multicenter RCT from 2004 found reduced mortality at 36 weeks of gestation but no significant effects on other clinical outcomes or oxygenation in infants <32 weeks of gestation (4). Finally, a study of beractant administered at 100 mg/kg vs. poractant at 200 mg/kg indicated short-term respiratory benefits with higher surfactant doses (more extubated patients at 48 and 72 h) (5).

Another factor under review was the adherence of surfactant dose to the recommended 200 mg/kg. In Poland, a 200-mg/kg dose is prescribed in most neonatal centers. Nonetheless, some degree of deviation from the target dose could be observed. This was most likely attributable to the prescription of full vials, which caused the dose of 200 mg/kg to often not be reached. In daily practice, the actual dose is frequently rounded down, particularly in more mature newborns with higher birth weight and better postnatal condition. We found this rounding down to be strongly associated with patients' maturity. While in neonates <26 weeks of gestation, the doses were generally equal to the recommended doses, the median underdosage in patients > 32 weeks exceeded 20%. A similar trend was found in the 2018 retrospective study by Boix et al. (2), in which the dose per kg increased with decreasing gestational age.

So far, only a few publications have described the exact doses of surfactant given, and they have reported underdosing. In a Spanish study from 2016, the mean initial dose of poractant alfa was 145.8 mg/kg (range 55–266 mg/kg) in a cohort of 119 infants with a mean gestational age of 30 weeks (19). Only 28 (23.5%) infants in this group received the dose of 200 mg/kg. In the already mentioned, more recent multicenter study of 206 infants from four tertiary neonatal units in Spain, the mean poractant dose (173.9 ± 37.3 mg/kg) was almost the same as that in our pooled cohort of 994 infants (170.2 ± 41 mg/kg) (2). Retrospective data from a French study showed a mean first surfactant dose of 188 mg/kg, and undertreated patients accounted for ~25% of the cohort (3). In surveys carried out in Europe, median values of the first dose of poractant were reported at 168–170 mg/kg (20, 21). Moreover, several European papers published between 2011 and 2015 reported a protocol-guided poractant alfa dose of 100 mg/kg (22, 23) or 100 mg/kg rounded to full vials (24). It appears that despite recommendations, the problem of underdosing in daily practice is not rare in European countries.

Poractant dose <200 mg/kg remains a risk factor for therapy failure not only with the traditional mode of administration (with intubation) but also with minimally invasive surfactant therapy (MIST), as was reported in a recent Dutch trial that enrolled 185 infants <32 weeks of gestation. Patients who failed MIST were at increased risk of severe intraventricular hemorrhage, indicating the extrapulmonary consequences of suboptimal treatment (16).

Surfactant overtreatment has also been observed in our cohort, although much less often, in just 32 (3.2%) infants. The maximum recorded surfactant dose in the studied cohort was 343 mg/kg, while in other trials, it was up to 400 mg/kg (3). While overtreatment does not seem to be a widespread problem, caution should be taken, as the consequences of the application of excessive doses remain unknown.

Our results should be interpreted with caution, as the analysis was based on collated data from three studies with different enrollment criteria. Additionally, we evaluated only the outcomes that were reported in all three studies. Another limitation is the lack of information on the precise criteria for surfactant retreatment across the three pooled trials. The observational character of the studies precluded the protocol-imposed conditions for surfactant retreatment, which, in this situation, followed the general guidance provided by the European RDS guidelines (“*ongoing evidence of RDS such as persistent high oxygen requirement and need for MV*”) (1). However, of the 139/994 infants who required surfactant redosing, most of the retreatments (78%) were associated with therapy escalation to MV. Only 31 (3%) infants were redosed due to persistently high oxygen requirements. Although the “*persistent high oxygen requirements*” may have been viewed differently from one center to another in these 3% of newborns, it does not seem to have had a major impact on the outcome of the study.

At the same time, we are presenting one of the most up-to-date European datasets regarding surfactant therapy based on a relatively large cohort. In addition, unlike most previous reports investigating the role of surfactant dosing (2, 12, 17), the majority of patients in the studied cohort were treated using LISA.

Despite the evolution of RDS management toward the more frequent use of noninvasive modalities of respiratory support and less invasive methods of surfactant administration, the benefits of high surfactant doses remain evident. The dose of 200 mg/kg poractant alfa reduces the need for invasive ventilation and retreatment. Further attention should be given to underdosing, particularly in more mature newborns, in whom underdosing is more prevalent.

## Data Availability Statement

The raw data supporting the conclusions of this article will be made available by the authors, without undue reservation.

## Ethics Statement

All three studies obtained prior approval from the Ethics Committee of Warsaw Medical University in accordance with the affiliation of the study principal investigator. Parents/legal guardians gave written consent to all diagnostic and therapeutic procedures in compliance with local legislation and practices.

## Author Contributions

All authors contributed equally to the design of the study, interpretation of findings, preparation of the initial draft of the manuscript, and all revisions. All contributors have read and accepted the final version of the manuscript.

## Conflict of Interest

BK-O received speaker honoraria from Chiesi Poland and a travel grant for participation in a scientific conference. TS received honoraria from Chiesi Poland for lecturing and participation on advisory boards. RH is employed by Chiesi Poland, the sponsor of the analyzed studies.
